# Supplementary material for: Association between NLRP3 rs10754558 and CARD8 rs2043211 Variants and Susceptibility to Chronic Kidney Disease
Source: Int J Mol Sci. 2023 Feb 20;24(4):4184. doi: 10.3390/ijms24044184 (PMC9963401; doi:10.3390/ijms24044184)
Supplement: Supplementary file 1 [file ijms-24-04184-s001.zip › ijms-2104541-supplementary.pdf]

Table S1. Clinical characteristics of KTRs, Dialysis and CKD stage 3-5 patients.

	**KTRs** (n = 143)	**Dialysis** (n = 126)	**CKD** (n = 34)
Age (years)	55.87 ± 12.23	66.51 ± 15.75	71.97 ± 9.99
Sex (Males)	92 (64.34)	83 (65.87)	24 (70.59)
Creatinine (mg/dl)	1.83 ± 1.30	8.84 ± 3.50	2.17 ± 0.79
eGFR (ml/min)	50.90 ± 25.69	9.19 ± 15.79	31.85 ± 11.66
Glucose (mg/dl)	95.02 ± 31.32	114.13 ± 42.22	127.14 ± 41.54
Urea (mg/dl)	44.98 ± 28.58	61.79 ± 20.38	82.15 ± 46.82
Uric Acid (mg/dl)	5.97 ± 1.70	5.85 ± 1.23	5.89 ± 1.35
Ca (mg/dl)	9.55 ± 0.86	9.31 ± 0.81	9.22 ± 0.61
Ph (mg/dl)	3.53 ± 1.01	4.78 ± 1.89	3.58 ± 0.86
Total Cholesterol (mg/dl)	193.66 ± 46.18	164.19 ± 48.49	171.77 ± 39.78
Tryglicerid (mg/dl)	179.72 ± 93.38	171.96 ± 96.41	177.59 ± 162.96
HDL Cholesterol (mg/dl)	54.26 ± 16.95	44.78 ± 17.05	41.62 ± 15.98
LDL Cholesterol (mg/dl)	103.15 ± 39.32	85.52 ± 40.69	86.60 ± 33.64
Ferritinemy (ng/ml)	104.00 (39.00 - 200.00)	298.50 (63.50 - 598.25)	49.50 (31.75 - 96.25)
CRP (mg/l)	5.00 (1.40 - 10.77)	7.18 (1.10 - 14.33)	0.60 (0.20 - 6.95)
serum Albumin (gr/dl)	3.93 ± 0.48	3.31 ± 0.50	4.18 ± 0.77
Hgb (gr/dl)	12.86 ± 1.72	11.58 ± 1.83	12.62 ± 2.12
Type 2 Diabetes	17 (11.89)	30 (23.81)	16 (47.06)
Hypertension	114 (79.72)	81 (64.29)	14 (41.18)
Cause CKD (%)			
Diabetic Nephropathy	6 (4.20)	28 (22.22)	14 (41.18)
Glomerular	95 (66.43)	10 (7.94)	14 (41.18)
Other	15 (10.49)	7 (5.56)	2 (5.88)
Unknown	27 (18.88)	81 (64.29)	0 (0.00)
NLRP3 rs10754558 (%)			
CC	33 (23.08)	33 (26.19)	11 (32.35)
CG	21 (14.69)	17 (13.49)	6 (17.65)
GG	89 (62.24)	76 (60.32)	17 (50.00)
CARD8 rs2043211 (%)			
AA	23 (16.08)	12 (9.52)	4 (11.76)
AT	25 (17.48)	62 (49.21)	12 (35.29)
TT	95 (66.43)	52 (41.27)	18 (52.94)

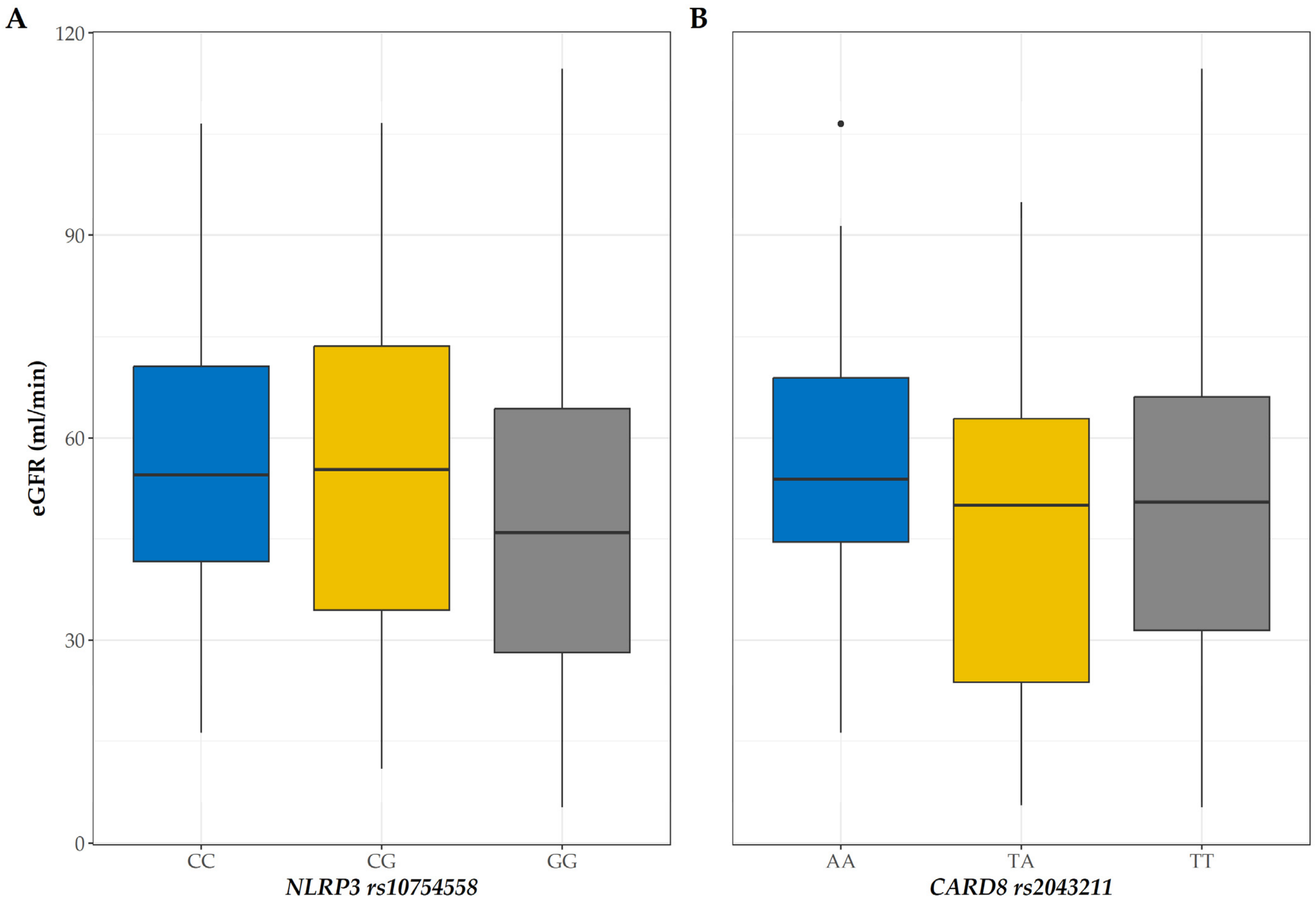

Figure S1. Association between eGFR levels and genotypes in KTRs.
